# A machine learning approach for gait speed estimation using skin-mounted wearable sensors: From healthy controls to individuals with multiple sclerosis

**DOI:** 10.1371/journal.pone.0178366

**Published:** 2017-06-01

**Authors:** Ryan S. McGinnis, Nikhil Mahadevan, Yaejin Moon, Kirsten Seagers, Nirav Sheth, John A. Wright, Steven DiCristofaro, Ikaro Silva, Elise Jortberg, Melissa Ceruolo, Jesus A. Pindado, Jacob Sosnoff, Roozbeh Ghaffari, Shyamal Patel

**Affiliations:** 1 MC10, Inc., Lexington, Massachusetts, United States of America; 2 Department of Biomedical Engineering, University of Vermont, Burlington, Vermont, United States of America; 3 Motor Control Research Laboratory, University of Illinois at Urbana-Champaign, Champaign, Illinois, United States of America; National Chiao Tung University College of Biological Science and Technology, TAIWAN

## Abstract

Gait speed is a powerful clinical marker for mobility impairment in patients suffering from neurological disorders. However, assessment of gait speed in coordination with delivery of comprehensive care is usually constrained to clinical environments and is often limited due to mounting demands on the availability of trained clinical staff. These limitations in assessment design could give rise to poor ecological validity and limited ability to tailor interventions to individual patients. Recent advances in wearable sensor technologies have fostered the development of new methods for monitoring parameters that characterize mobility impairment, such as gait speed, outside the clinic, and therefore address many of the limitations associated with clinical assessments. However, these methods are often validated using normal gait patterns; and extending their utility to subjects with gait impairments continues to be a challenge. In this paper, we present a machine learning method for estimating gait speed using a configurable array of skin-mounted, conformal accelerometers. We establish the accuracy of this technique on treadmill walking data from subjects with normal gait patterns and subjects with multiple sclerosis-induced gait impairments. For subjects with normal gait, the best performing model systematically overestimates speed by only 0.01 m/s, detects changes in speed to within less than 1%, and achieves a root-mean-square-error of 0.12 m/s. Extending these models trained on normal gait to subjects with gait impairments yields only minor changes in model performance. For example, for subjects with gait impairments, the best performing model systematically overestimates speed by 0.01 m/s, quantifies changes in speed to within 1%, and achieves a root-mean-square-error of 0.14 m/s. Additional analyses demonstrate that there is no correlation between gait speed estimation error and impairment severity, and that the estimated speeds maintain the clinical significance of ground truth speed in this population. These results support the use of wearable accelerometer arrays for estimating walking speed in normal subjects and their extension to MS patient cohorts with gait impairment.

## Introduction

Gait impairment is a common phenotype prevalent in patients suffering from a variety of neurological disorders including multiple sclerosis (MS). In MS, inflammation in the brain and chronic demyelination cause a degeneration in axon conduction which manifests as the progressive clinical disability characteristic of the disorder [1]. One such group of symptoms result in mobility (i.e. gait) impairment [2], which has been identified by altered spatio-temporal gait parameters including slower gait speed [3]. Mobility impairments have been strongly correlated with progression of MS [4–6] and risk for adverse outcomes including falls [7], reduced quality of life [8], and increased caregiver burden [8]. For example, 41% of MS patients report some form of walking impairment, and 13% report that they are unable to walk beyond 2 days per week [8]. These mobility impairments have been shown to negatively affect both patient and caregiver quality of life [8]. Walking impairment has been observed early in the disease course of MS, and even in the absence of other, pyramidal, indicators of the disease [4]. For example, a study of 20 minimally impaired MS patients (Expanded Disability Status Scale—EDSS = 0.0–2.5: N = 10 Pyramidal Functional Systems—PFS = 0.0, N = 10 PFS = 1.0) and 20 age- and gender-matched controls revealed a significant reduction in walking speed with MS diagnosis [4]. This trend continues with disease progression, as MS patients in the PFS = 1.0 group demonstrated significantly reduced walking speed and stride length when compared with the PFS = 0.0 group [4]. These results extend beyond this minimally impaired MS population, with cross-sectional studies demonstrating that increases in MS-related disability are negatively correlated with walking speed [9] and endurance [5,10] across the spectrum of mobility impairment levels (EDSS 0.0–6.5).

This evidence points toward the importance of walking speed as a parameter for characterizing mobility impairments in the MS population. However, typical clinic-based assessments of walking speed (i.e., 6 minute walk test, timed 25 foot walk, etc.) might not accurately represent a patient’s walking performance outside the clinic [11]. Moreover, even if these functional tests were administered at every visit, a measurement frequency of 3–6 months does not provide sufficient granularity to track intervention efficacy, nor does it enable rapid tailoring of intervention course to an individual to yield optimal response. One potential solution to this problem is to instrument MS patients with wearable motion tracking devices outside of the clinic to better understand their natural walking speed patterns, and how they change over more granular timescales.

A number of recent studies have applied wearable inertial sensors, including accelerometers and/or angular rate gyroscopes, for tracking walking speed outside of the clinical setting [12]. These techniques can be categorized as i) direct integration [13–17], ii) human gait model [18–22], or iii) machine learning (ML) models [23–26]. The direct integration and human gait model techniques are capable of providing excellent results (root-mean-square-error (RMSE) of 0.06 m/s [22]), but they require an angular rate gyroscope, which increases the cost of the wearable device and significantly reduces battery life. While these limitations do not affect deployment during constrained clinical tests, they do hinder translation to estimating walking speed during ambulation outside the clinic.

The ML model technique for estimating walking speed utilizes supervised learning to train a statistical model relating measurements of walking kinematics with speed. In practice, walking kinematics are often measured by one (or several) body-worn accelerometer(s) and decomposed into a series of parameters (features) that summarize the kinematics. These parameters are also sometimes combined with anthropometric information including subject height, weight, and leg segment lengths. A model that relates these kinematic and anthropometric parameters to walking speed is then ‘learned’ from a series of training examples collected at a variety of walking speeds and from a variety of subjects. The hypothesis is that larger ‘training sets’ yield models that more accurately capture the true relationship between the measured kinematics (and anthropometric characteristics) and speed. Several studies have employed this technique to translate data from accelerometer-only and accelerometer and gyro based wearable devices into estimates of walking speed [23–26]. One study, that employs a belt-worn accelerometer, found that a support vector regression model produced superior results to output from human gait model based approaches that exploit relationships between stride frequency and step length [23]. They reported a coverage probability (95% confidence interval), relative to a gold standard perambulator, of 0.46 (0.12–0.70) at the 0.1 m/s error level from a study of 17 normative subjects walking over ground at 4 self selected speeds [23]. However, 8 subjects from their training set were included in their testing set, which could artificially improve this level of agreement. Another study utilized treadmill walking data collected from 20 subjects walking at speeds ranging from 0.7 to 1.7 m/s to assess a mobile phone (located in the subject’s pocket) based algorithm for estimating walking speed [25]. The authors report an RMS error of 0.12 m/s for an accelerometer-only feature set and a leave-one-subject-out error estimation approach. While existing studies demonstrate that the accuracy of the direct integration and human gait model techniques is superior to that of the ML model technique, the accelerometer-only measurement requirements drastically reduce the barrier to deployment outside of the clinic. Accordingly, it is important to understand if there are device locations, or combinations of locations, that can further improve walking speed estimation accuracy using this approach.

Walking speed is a primary indicator of outcome in clinical research and practice involving MS patients [27]. New wearable technology, and the ML model based approaches described above, could provide a means for characterizing walking speed in more naturalistic environments. Traditionally, an ML model for estimating walking speed in patients with MS would require a training dataset sampled from a population of patients with varying levels of MS-induced gait impairments walking at a variety of speeds. However, collection of these data puts patients at heightened risk for falls which could yield significant physical and emotional injury, negatively impacting ambulation (and therefore quality of life [8]) for weeks, months, and years following the accident. One potential solution, which would obviate the need for collection of training data on patients with MS-induced gait impairment, is to train ML models for walking speed on data from healthy subjects. However, it is then important to explore how these models developed on normal subjects generalize to populations with gait impairments, and in the case of MS, how the model performs as a function of impairment severity. For example, one study explored the performance of walking speed estimations provided by a belt-worn commercial activity tracking device, and their model trained on subjects without neurological disorders, in a sample of 51 MS patients spanning the disability spectrum (EDSS 2.0–6.5) [28]. They found that estimations were accurate for subjects with mild impairment (EDSS 2.0–3.5), but the accuracy degraded considerably for moderately (EDSS 4.0–5.5) and severely (EDSS 6.0–6.5) impaired subjects.

Here we develop a ML-based technique for estimating walking speed using a new, conformal wearable device. We utilize accelerometers from multiple body locations to characterize gait speeds during treadmill walking in normative subjects (control), and during treadmill walking for a population of subjects diagnosed with multiple sclerosis (MS). We further investigate how different combinations of device locations influence estimation accuracy, and if estimated speeds maintain the clinical relevance of the ground truth speed values.

## Methods

Research conducted at the University of Illinois at Urbana-Champaign (UIUC) was approved by the UIUC Institutional Review Board (IRB Protocol Number: 15853). Research at MC10, Inc. and UIUC were conducted according to the principles expressed in the Declaration of Helsinki. Written, informed consent was obtained from each subject prior to participation.

### A. Study design

The BioStamp Research Connect^™^ system (BioStampRC—MC10, Inc., Lexington, MA) was used to design two experimental protocols, one for a sample of normative subjects (Protocol A) and one for a sample of MS patients and healthy controls (Protocol B). Protocol A included a series of 6-minute treadmill walking tests executed at 5 predefined speeds (0.5, 0.75, 1.0, 1.25, and 1.5 m/s). Protocol B included a variety of tests employed during clinical assessments of mobility for MS patients including a Postural Control Test (PCT) (two repeated bouts of eyes open, eyes closed, and eyes open standing on a foam pad), and the Six Minute Walk Test (6MWT) completed at three self-selected speeds (slow, comfortable, fast). Speeds for the 6MWT were determined for each subject from a test of comfortable walking over a Zeno Walkway (ProtoKinetics, Havertown, PA). The BioStampRC^™^ Investigator Application was utilized to log the total duration and average walking speed for each activity, where applicable.

### B. Participants

For Protocol A, a sample of 10 subjects (sex: 4F/6M, age: 22–35 yrs, height: 1.6–1.8 m, weight: 61.2–99.3 kg) was recruited from a population of subjects with no known gait impairments. For Protocol B, a sample of 37 subjects, 30 diagnosed with MS (sex: 21F/9M, age: 29–74 yrs, height: 1.5–1.8 m, weight: 43.1–136.0 kg, EDSS_SR_: 0.0–7.0, MSWS: 0.20–0.95) and 7 healthy controls (sex: 3F/4M, age: 37–71 yrs, height: 1.6–1.8 m, weight: 64.4–102.5 kg), was recruited from the community surrounding the University of Illinois at Urbana-Champaign. Informed written consent was collected from each subject prior to participation in either protocol.

### C. Protocol

Following collection of informed consents, subjects in Protocol A were instrumented with BioStampRC Sensors and instructed to walk on a treadmill. The treadmill speed was ramped until the desired walking speed was achieved for a given test (see S1 Appendix for validation of treadmill reported speeds). Once the treadmill had reached steady state, the study administrator used the BioStampRC Investigator Application to indicate the beginning and end of the walking test, and to record the walking speed. This sequence was repeated until tests for the five walking speeds had been completed.

For Protocol B, subjects completed a verbal health history and physical activity readiness questionnaire (PAR-Q) during a phone screen and, if found to meet the inclusion criteria, were scheduled for an onsite visit at the Motor Control Research Laboratory at the University of Illinois at Urbana-Champaign. During the onsite visit, subjects provided written informed consent, and completed questionnaires including a Patient Demographics Survey, the Twelve Item MS Walking Scale (MSWS), the Self-Reported Expanded Disability Status Scale (EDSS_SR_), and the Fall, Trips, and Slips 6 Month Survey. Subjects were then instrumented with BioStampRC Sensors and completed a series of functional tests designed to assess balance and mobility impairments in MS patients including the PCT, the Timed 25 Foot Walk Test, the Timed Up and Go, and the 6MWT. Herein, we consider data from the PCT and the 6MWT in particular. For the PCT, subjects were tested twice in each of three conditions (eyes open, eyes closed, and eyes open standing on a foam pad) for 30 seconds. The study administrator used the BioStampRC Investigator Application to indicate the beginning and end of each PCT, during which the subject’s speed was assumed to be 0 m/s. For the 6MWT, subjects were tested at three different speeds (comfortable, 20% above comfortable and 20% below comfortable). The study administrator used the BioStampRC Investigator Application to indicate the beginning and end of each 6MWT. Immediately after indicating the beginning of the test, subjects began to walk on the treadmill while the administrator increased the speed until the desired walking speed was achieved. The steady-state treadmill speed for each test was recorded on a test datasheet, copies of which were provided for each subject for analysis.

### D. Instrumentation

**Fig 1:**
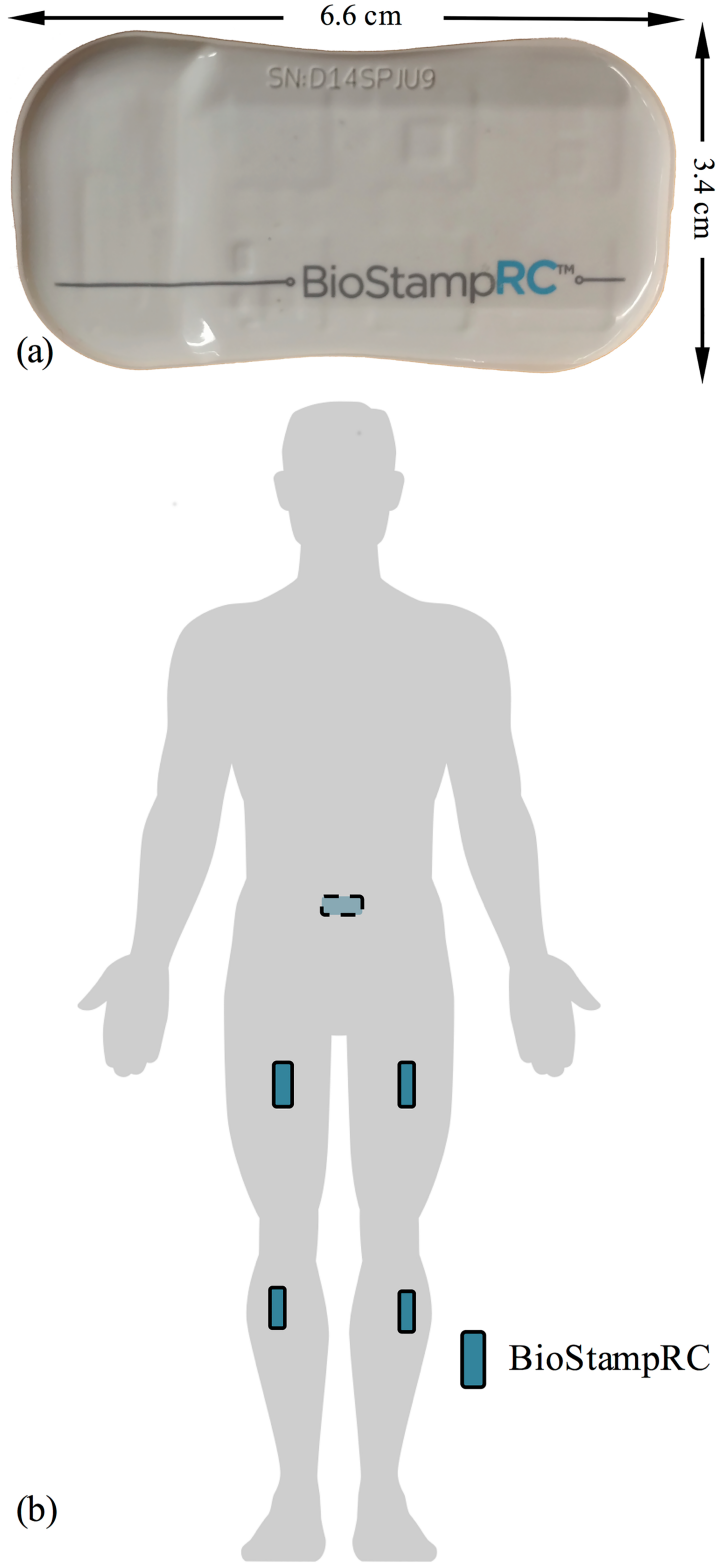
BiostampRC sensors are skin-mounted, conformal devices that can be adhered at multiple locations on the body. (A) BioStampRC skin-mounted, conformal motion sensor. (B) Anatomical locations where devices were adhered to the skin.

Subjects in Protocols A and B were instrumented with five BioStampRC Sensors (See Fig 1A), affixed to the skin at the locations illustrated in Fig 1B (sacrum, bilateral thigh, bilateral shank). Each sensor was configured to log accelerometer (measurement range—Protocol A: +/- 8G, Protocol B: +/- 4G) data to onboard flash memory at a rate of 50 Hz. Each accelerometer sample, from each device, was recorded with a corresponding absolute time stamp thus allowing direct comparison of data across devices. Following the completion of all data collection activities for each subject, data were downloaded from the sensors using the Investigator Application, and uploaded to the BioStampRC Investigator Portal in the MC10 cloud for further analysis.

### E. Pre-processing and feature extraction

BioStampRC Sensors were secured to the body such that the axis orthogonal to the plane of the device (Z) was directed roughly in the anterior (thigh, shank) or posterior (sacrum) directions. The accelerometer data from each axis is low-pass (12 Hz cutoff [29]) and band-pass (0.25–12 Hz cutoff) filtered, yielding a total of six time series, from each device, for analysis. The prescribed device placement constrains the direction of the Z-axis, but the remaining axes (X,Y) are largely arbitrary. To remove further dependence on device orientation, we take the vector magnitude of accelerometer signals in the X and Y directions (XY = sqrt(X^2^ + Y^2^)), yielding two time series, Z and XY, for each filtering approach (band-pass, low-pass), for further analysis. These time series are segmented into 5-second non-overlapping windows. Each window represents one sample or observation. Thus, a single 6MWT yields 72 samples or observations for model training. Data from each window are parameterized by a set of time and frequency domain features [30] to inform estimates of walking speed. Time domain features include mean value, range, kurtosis, signal entropy, range of auto-covariance and correlation coefficient (XY with Z). Frequency domain features include spectral entropy, value and magnitude of dominant frequency, and ratio of energy in the dominant frequency to the band-limited frequency spectrum. A total of 32 features are extracted from each window of data, for each device location.

### F. Model training and performance evaluation

To estimate walking speed from these accelerometer-based features, we define support vector regression models [23] (*f*) of the form

$$s=f\left(\vec{a}\right)$$ (1)

where *s* is the average walking speed during a 5-second period of time and $\vec{a}$ is an M-element vector composed of 32 time and frequency domain features extracted from the corresponding 5-second window of accelerometer data, from each of the desired sensor locations. We train models for seven combinations of the sacrum, right thigh, and right shank locations in Python using Scikit-Learn [31] and a supervised-learning approach. The training dataset consists of data from the subjects of Protocol A (N = 10) and the control subjects of Protocol B (N = 7), which together yield a total of 17 normal subjects for model development and analysis (data for each device location combination available in S1 File). The treadmill speeds from these tests provide ground-truth speed values (*s*) that enable training of walking speed models (*f*). To establish the accuracy of these models on normal subjects, we employ a leave-one-subject-out approach, where a walking speed model is trained on 16 subjects, and used to estimate speed during tests completed by the remaining subject. This process is repeated until all subjects have been left out of the training set. To establish model performance on impaired subjects, we train walking speed models using all 17 normal training subjects and estimate speed during the 6MWTs completed by each of the 30 subjects diagnosed with MS (data for each device location combination from each MS subject available in S2 File). For both the normal and impaired tests, we compute the median of the estimated speeds for comparison to the ground-truth values reported by the treadmill. This approach yields a total of 114 observations (5 tests from each subject in Protocol A, 9 tests from each control subject in Protocol B, and one extra 6MWT completed by a subject in Protocol B) for the analysis of performance on normal gait, and 91 observations (one subject completed an extra 6MWT) for the analysis of performance on impaired gait (data for each device location combination available in S3 File).

To analyze the performance of the walking speed estimations for normal and impaired subjects, we report the root-mean-squared-error (RMSE), the Bland-Altman limits of agreement (LOA), and the slope (*m*) and intercept (*b*) of the following linear model

$$y=m\cdot \hat{y}+b$$ (2)

where *y* corresponds to the truth values, and $\hat{y}$ corresponds to the associated estimates (median speed from each walking test). The Bland-Altman limits of agreement describe the 95% confidence interval for the difference between measurement techniques which provides an indication of the minimal detectable difference in the estimated speed [32]. The intercept (*b*) indicates the systematic difference between measurement techniques, and the slope (*m*) indicates how closely one measurement technique is able to detect changes in the other.

We examine error in the walking speed estimates as a function of impairment level in two ways. First we examine the relationship between error and MSWS and EDSS_SR_ scores for each subject by considering the Pearson product moment correlation coefficient (r). We then divide the MS patients into Mild (N = 14), Moderate (N = 10), and Severe (N = 6) impairment groups and compare the error in estimated speed within each group. Subjects were assigned to groups based on their comfortable walking speed during the Timed 25 Foot Walk Test: Mild—speed > = 1.1 m/s, Moderate– 0.7 m/s < speed < 1.1 m/s, and Severe—speed < = 0.7 m/s [33].

Finally, we establish concurrent validity in the context of MS, by examining the relationship between estimated and ground truth walking speeds sampled from the comfortable 6MWT of Protocol B and indicators of mobility impairment and fall risk. Specifically, the Pearson product moment correlation coefficient is used to characterize the relationship between walking speed and MSWS and EDSS_SR_ scores, and the Mann-Whitney U test is used to test for a significant difference in walking speed between subjects who reported a fall in the six months prior to the test and those who did not. For all statistical analyses, significance is assessed at the α = 0.05 level.

## Results

We trained support vector regression [23] models to estimate walking speeds based on feature sets extracted from a combination of devices secured to the sacrum, thigh and shank. The 17 subjects with normal gait exhibited speeds ranging from 0.0 to 1.7 m/s (0.0 m/s speeds from the PCT tests of Protocol B). Results from the leave-one-subject-out error estimation for each of the seven possible device location combinations are shown in Table 1. For each device location combination, we report the RMSE, LOA, and the regression analysis of Eq (2).

**Table 1:** Error metrics for treadmill walking data from healthy subjects for 7 device location combinations.

Device Locations	RMSE (m/s)	LOA (m/s)	Best Fit Line
Sacrum	0.15	(-0.31, 0.29)	y = 0.97$\;\hat{y}$+0.03
Thigh	0.15	(-0.30, 0.27)	y = 0.96$\;\hat{y}$+0.04
Shank	0.13	(-0.26, 0.25)	y = 0.98$\;\hat{y}$+0.02
Sacrum, Thigh	0.16	(-0.34, 0.29)	y = 0.98$\;\hat{y}$+0.04
Sacrum, Shank	0.13	(-0.26, 0.25)	y = 1.01$\;\hat{y}$+0.00
Thigh, Shank	0.11	(-0.23, 0.21)	y = 0.98$\;\hat{y}$+0.03
Sacrum, Thigh, Shank	0.12	(-0.25, 0.22)	y = 1.00$\;\hat{y}$+0.01

Metrics include root-mean-squared-error (RMSE), Bland-Altman limits of agreement (LOA), and best fits from the regression analysis.

**Fig 2:**
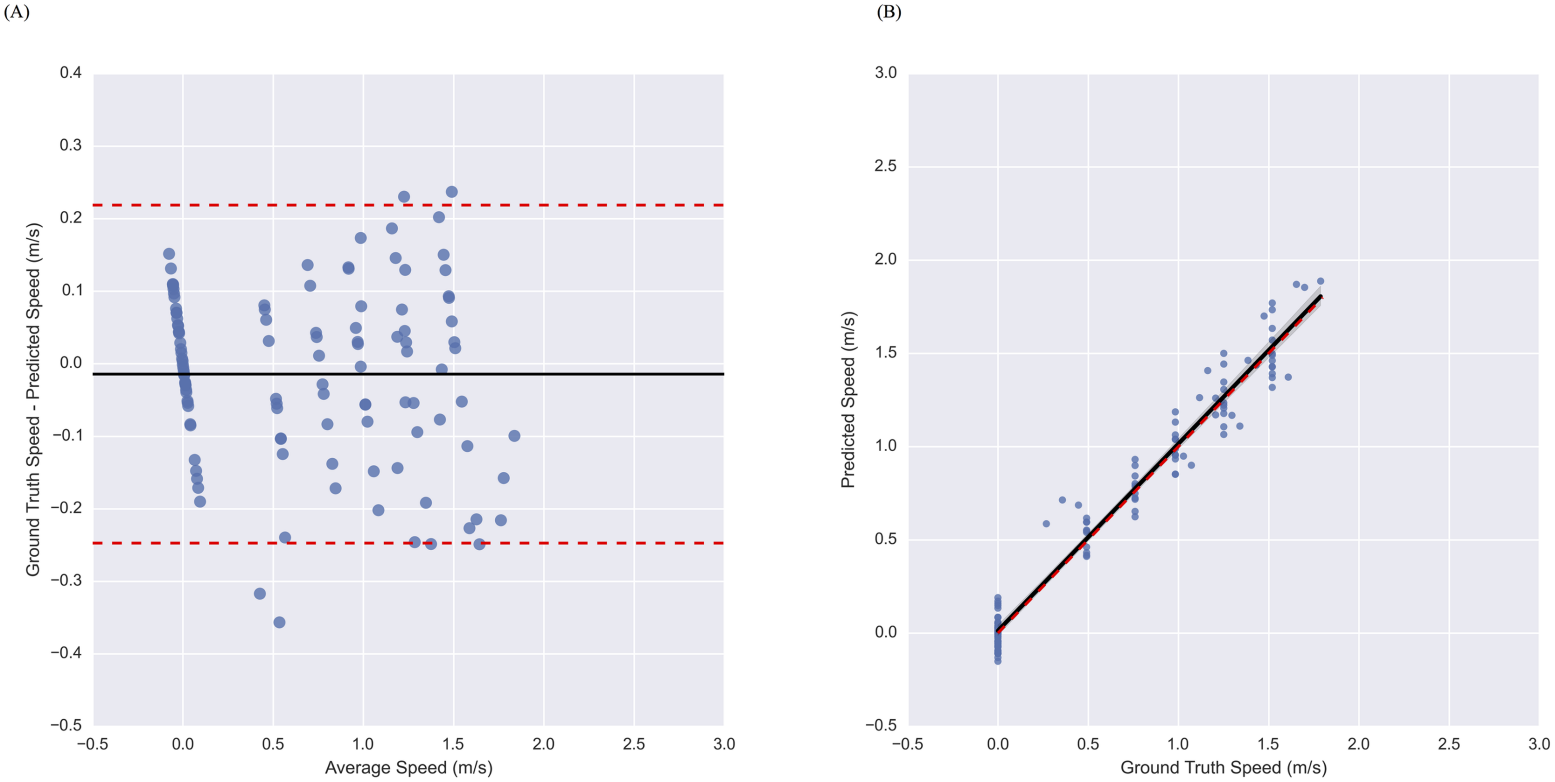
Performance of model using sacrum, thigh, and shank device locations on treadmill data from healthy subjects. Bland-Altman (A) and regression (B) plots illustrating the performance of the accelerometer-based model for estimating walking speed on a treadmill. As illustrated in the plots, the model produces unbiased estimates of speed with homoscedastic error.

We also considered the Bland-Altman and regression plots for the Sacrum, Thigh, Shank model of Table 1 in Fig 2A and 2B, respectively. Each point of the Bland-Altman plot represents a single walking speed test, where the y-coordinate is the error in the speed estimate and the x-coordinate is the average between the estimated and true speed. The solid black line indicates mean error in the speed estimate, and the dashed red lines indicate the limits of agreement (95% confidence interval). Similarly, each point of the regression plot represents a single walking speed test, where the y-coordinate is the estimated speed and the x-coordinate is the true speed. The line of best fit is plotted as a solid black line, the 95% confidence region for the regression is shaded in grey (estimated via bootstrap), and a dashed red line with unit slope and zero intercept, is shown for reference.

We now assess the accuracy of walking speed estimations for patients with gait impairments from models trained on 17 subjects without gait impairments. Table 2 reports error metrics for models trained on data from each of the seven device location combinations for 30 MS patients, with varying levels of gait impairment. Actual walking speeds during these tests ranged from 0.13 to 1.88 m/s.

**Table 2:** Error metrics for treadmill 6MWT data from MS patients for 7 device location combinations.

Device Locations	RMSE (m/s)	LOA (m/s)	Best Fit Line
Sacrum	0.12	(-0.25, 0.22)	y = 0.96$\;\hat{y}$+0.05
Thigh	0.16	(-0.36, 0.23)	y = 1.93$\;\hat{y}$+0.12
Shank	0.16	(-0.32, 0.31)	y = 0.89$\;\hat{y}$+0.10
Sacrum, Thigh	0.13	(-0.25, 0.26)	y = 1.02$\;\hat{y}$-0.02
Sacrum, Shank	0.14	(-0.26, 0.27)	y = 0.94$\;\hat{y}$-0.05
Thigh, Shank	0.14	(-0.3, 0.25)	y = 0.95$\;\hat{y}$+0.06
Sacrum, Thigh, Shank	0.14	(-0.27, 0.27)	y = 0.99$\;\hat{y}$+0.01

Metrics include the root-mean-squared-error (RMSE), Bland-Altman limits of agreement (LOA), and the best fit line from the regression analysis.

**Fig 3:**
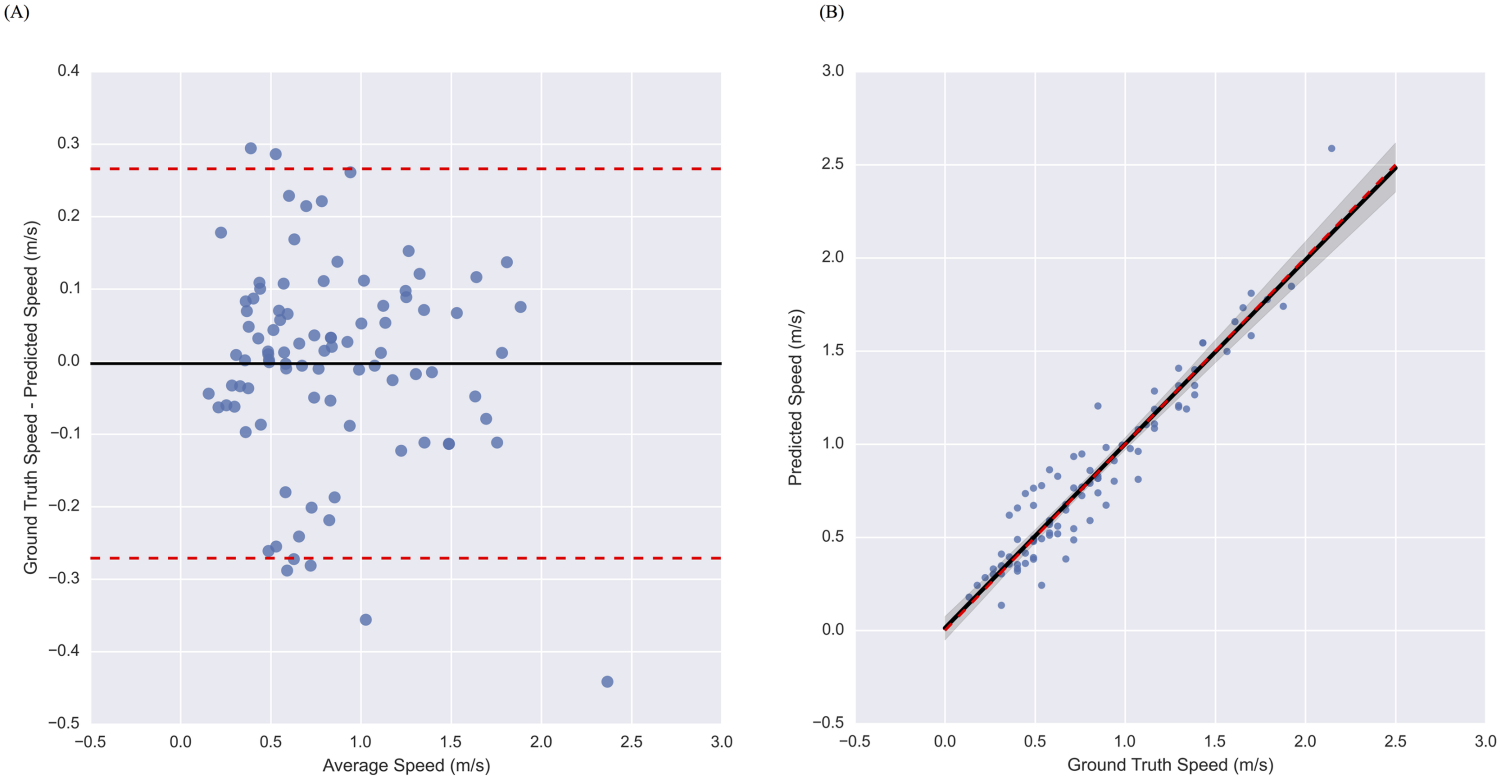
Performance of model using sacrum, thigh, and shank device locations on treadmill data from MS patients. Bland-Altman (A) and regression (B) plots illustrating the performance of the accelerometer-based model for estimating walking speed in MS patients. As illustrated in the plots, the model produces unbiased estimates of speed with slightly higher variance at slower speeds, despite being trained on data from subjects with normal gait.

We further examine the error in walking speed estimates for the Sacrum, Thigh, Shank model of Table 2 by considering the Bland-Altman and regression plots shown in Fig 3A and 3B, respectively.

To better understand the relationship between accuracy and impairment level, we report the RMSE and LOA for the Mild, Moderate, and Severe subject groups, and the Pearson product moment correlation coefficient between estimation error and EDSS_SR_ and MSWS scores, for each of the seven device location combinations, in Table 3.

**Table 3:** Error metrics by MS impairment groups and Pearson product moment correlation between error and EDSS_SR_ and MSWS scores.

Device Locations	Mild		Moderate		Severe		Correlation of error with EDSS_SR_	Correlation of error with MSWS
	RMSE (m/s)	LOA (m/s)	RMSE (m/s)	LOA (m/s)	RMSE (m/s)	LOA (m/s)		
Sacrum	0.13	(-0.24, 0.25)	0.12	(-0.27, 0.20)	0.11	(-0.24, 0.17)	-0.32	-0.28
Thigh	0.17	(-0.37, 0.26)	0.14	(-0.31, 0.18)	0.18	(-0.40, 0.22)	-0.23	-0.12
Shank	0.16	(-0.29, 0.33)	0.14	(-0.23, 0.30)	0.21	(-0.45, 0.19)	-0.38^+^	-0.29
Sacrum, Thigh	0.12	(-0.24, 0.22)	0.12	(-0.20, 0.27)	0.16	(-0.32, 0.32)	0.02	0.19
Sacrum, Shank	0.14	(-0.23, 0.29)	0.10	(-0.12, 0.22)	0.19	(-0.39, 0.12)	-0.39^+^	-0.20
Thigh, Shank	0.14	(-0.29, 0.27)	0.11	(-0.21, 0.23)	0.18	(-0.40, 0.18)	-0.35	-0.18
Sacrum, Thigh, Shank	0.14	(-0.28, 0.28)	0.10	(-0.13, 0.22)	0.17	(-0.37, 0.18)	-0.28	-0.05

Error metrics include RMSE and LOA. Statistical significance at the α = 0.05 level indicated with ^+^.

**Fig 4:**
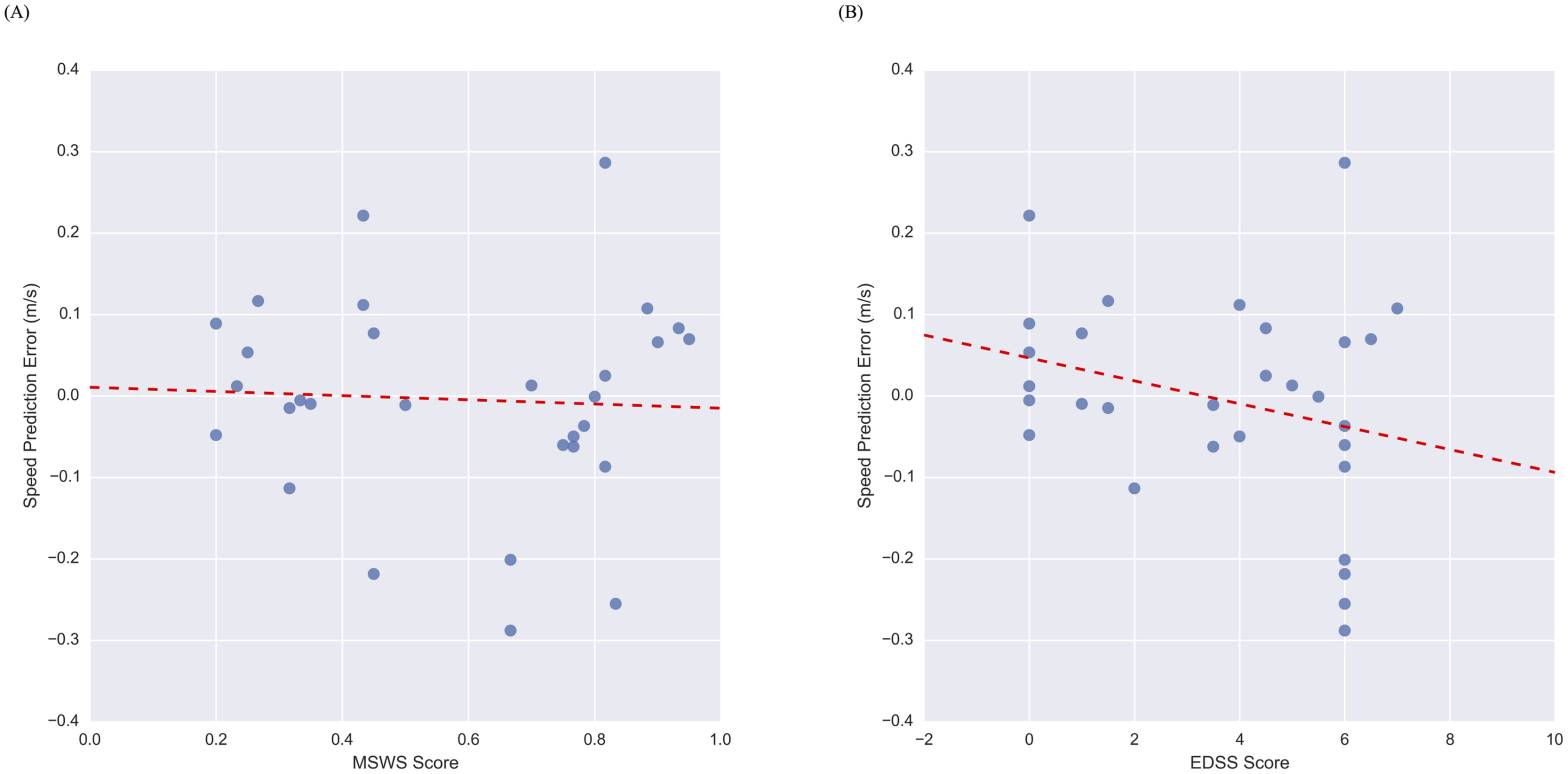
Scatter plots showing the relationship between speed estimation error and MSWS and EDSS_SR_. Speed estimation error vs. MSWS score (A) and EDSS_SR_ score (B) from the sacrum, thigh, shank model. The dashed red line is a line of best fit, correlations between error and EDSS_SR_/MSWS scores are not significant.

We further examine the accuracy of walking speed estimations across impairment levels with the Sacrum, Thigh, Shank model in Fig 4A and 4B, where we plot speed estimation error versus MSWS and EDSS_SR_ scores, respectively. Each point in Fig 4A/4B, where the y coordinate is the speed estimation error and the x coordinate is the MSWS/EDSS_SR_ score, represents a single 6MWT. In each plot, the red dashed line is the best-fit line for the data.

Finally, we quantify the ability of each model to preserve the clinical relevance of the ground truth data by correlating estimated and ground truth walking speeds with MSWS and EDSS_SR_ scores. We also test for differences in speed between groups of subjects who reported a fall in the 6 months prior to the study (Fall) and those who did not (No Fall, none of the differences were statistically significant). Subjects in the Fall group are expected to have slower comfortable walking speeds than those in the No Fall group [7]. Results are presented in Table 4.

**Table 4:** Relationship between walking speed and MSWS score, EDSS_SR_ score, and fall history.

Device Locations	Correlation of speed with EDSS_SR_ score		Correlation of speed with MSWS score		Mean speed difference (m/s) between Fall and No Fall groups	
	Estimated	Ground Truth	Estimated	Ground Truth	Estimated	Ground Truth
Sacrum	**-0.75**	**-0.79**	**-0.84**	**-0.87**	-0.20	-0.22
Thigh	**-0.75**	**-0.79**	**-0.86**	**-0.87**	-0.19	-0.22
Shank	**-0.77**	**-0.79**	**-0.89**	**-0.87**	-0.21	-0.22
Sacrum, Thigh	**-0.76**	**-0.79**	**-0.88**	**-0.87**	-0.21	-0.22
Sacrum, Shank	**-0.72**	**-0.79**	**-0.87**	**-0.87**	-0.24	-0.22
Thigh, Shank	**-0.74**	**-0.79**	**-0.88**	**-0.87**	-0.23	-0.22
Sacrum, Thigh, Shank	**-0.73**	**-0.79**	**-0.88**	**-0.87**	-0.25	-0.22

Pearson product moment correlation coefficient was used to relate walking speed to MSWS and EDSS_SR_ scores. The difference in comfortable walking speed between fall groups in estimated and ground truth data is shown to relate speed with fall history. Statistical significance at the α = 0.01 level is indicated in bold.

## Discussion

We present a technique for estimating walking speed based on data from an array of wearable accelerometers, and conduct a detailed analysis of the influence of device placement combinations on estimation accuracy in a sample of subjects without gait impairments. We further explore how models trained on subjects without gait impairments perform in a sample of patients diagnosed with MS-induced gait abnormalities. In so doing we characterize estimation accuracy across impairment levels and device placement combinations. Finally, we establish clinical utility by comparing ground truth and estimated walking speeds to clinical assessments of impairment levels as quantified by the EDSS_SR_ and MSWS scales and if the subject has reported falling in the six months prior to their visit.

The results presented in Table 1 show high accuracy of the proposed technique for estimating walking speed in subjects without gait impairments across a variety of device location combinations. Generally, performance improves with both distal device locations and additional devices. For example, walking speeds estimated by a model trained on data from just the sacrum device has a RMSE of 0.15 m/s, with a 95% confidence interval for error of (-0.31, 0.29) m/s, the ability to quantify changes in speed to within 3%, and a systematic bias of 0.03 m/s. In contrast, the model trained on data from the shank device improves upon these error characteristics by estimating walking speed with a reduced RMSE (0.13 m/s), smaller 95% confidence interval for error (-0.26, 0.25) m/s, better ability to quantify changes in speed (within 2%), and a reduced systematic bias (0.02 m/s). This increase in performance is achieved by simply placing the BioStampRC Sensor to a different body location. The performance of these single-device models compare well with the best single-device approach reported in the literature that is able to quantify walking speed with an RMSE of 0.12 m/s (or 0.11 m/s after gps-based subject-specific calibration) [25]. Adding additional devices can further improve these results, provided that care is taken to avoid over-fitting. This is evidenced by the error characteristics for the model trained on data from the sacrum, thigh, and shank locations, which yields a RMSE of 0.12 m/s, a 95% confidence for the error of (-0.25, 0.22) m/s, is able to quantify changes in speed to within less than 1%, and systematically overestimates speed by only 0.01 m/s. The Bland-Altman and regression plots of Fig 2A and 2B, highlight the unbiased speed estimate provide by the three-device model, and demonstrate the homoscedasticity of the variance.

The results of Table 2 establish how the proposed modeling approach for estimating walking speed generalizes to subjects with MS-induced gait impairments, and illustrates how changes in the number and location of devices affects accuracy. Specifically, the results suggest the opposite trend observed in the non-impaired sample, namely that error increases with distal device placements. For example, a model trained on data from the sacrum device yields a RMSE of 0.12 m/s, 95% confidence interval for the error of (-0.25, 0.22) m/s, the ability quantify changes in speed to within 4%, and systematically overestimates speed by 0.05 m/s. In contrast, a model trained on data from the shank yields larger RMSE (0.16 m/s), wider limits of agreement (-0.32, 0.31), more limited ability to detected changes in speed (within 11%), and a larger systematic bias (0.10 m/s). This could be due to gait impairments manifesting as larger deviations in the kinematics of the distal body segments, where mass center (or sacral) kinematics need to be more tightly constrained to enable even severely impaired ambulation. Nevertheless, the inclusion of distal device locations with the sacrum, acts to alter the bias-variance characteristics of the estimation error. Namely, a model trained on data from the sacrum, thigh, and shank devices yields slightly higher variance as evidenced by increased RMSE (0.14 m/s) and wider limits of agreement (-0.27, 0.27), but reduces bias with improvements in the ability to quantify changes in speed (now within 1%) and the systematic bias (overestimate speed by only 0.01 m/s). The Bland-Altman and regression plots of Fig 3A and 3B, respectively, help to visualize the performance of this model across a wide range of walking speeds and the associated gait impairment levels. This model seems to introduce a slight heteroscedasticity, where the slowest speeds seem to elicit slightly larger error variance, however this could also be due to our dataset, which contains fewer observations at faster speeds.

To this end, we examine error characteristics within each impairment level (mild, moderate, severe) as a function of the device location combinations in Table 3. This analysis demonstrates that the sacrum is the single best location for estimating walking speed across impairment levels with very little difference between levels. Ultimately, this suggests that the proposed approach for estimating walking speed is able to generalize across gait impairments. This compares favorably to existing methods for estimating walking speed from accelerometer data. Specifically, a validation study of the actibelt^®^, a belt-worn accelerometer-based device, in a sample of 51 MS patients with gait impairments demonstrated that error in walking speed estimates differed significantly across impairment levels. For subjects with mild, moderate, and severe impairments, the reported mean (standard deviation) error in walking speed estimation was -0.02 (0.11), -0.10 (0.16), and -0.26 (0.12) m/s, respectively [28]. For comparison, the sacrum model presented herein yields mean (standard deviation) error in walking speed estimations of 0.01 (0.12), -0.04 (0.12), and -0.04 (0.11) m/s for subjects with mild, moderate, and severe gait impairments, respectively. This improvement in results across impairment groups could be due to the conformal, skin-mounted nature of the BioStampRC sensor (vs. the belt-worn actibelt^®^), or differences in the modeling approach and/or feature set employed for estimating speed. To further confirm the lack of relationship between error in the walking speed estimations and gait impairments, we correlate walking speed error with EDSS_SR_ and MSWS scores for models trained with each device location combination in Table 3. Estimation errors for none of the walking speed models show significant (α = 0.05) correlations with MSWS scores, and only 2 of the 7 models (Shank; Sacrum, Shank) show correlation with EDSS_SR_ score. These results further confirm the lack of relationship between error in the walking speed estimations and impairment level for many of the device location combinations. This is an important result, as it suggests that the proposed approach allows extension of models for estimating walking speed developed on data from normal subjects to MS subjects with gait impairments without introducing additional error that could potentially confound interpretation of the results.

Finally, we examine the relationship between the ground truth and estimated walking speeds during tests conducted at the comfortable walking speed, and clinical measures of impairment and fall history to assess if the proposed technique is able to capture the clinical significance of this important variable. Table 4 demonstrates that both ground truth and estimated speeds are significantly, and similarly, correlated with MSWS scores (estimated: *r* ≤ −0.84,*p* < 0.01, ground truth: *r* = −0.87,*p* < 0.01) such that walking speed decreases as MSWS score, and therefore walking impairment, increases. These results agree well with existing literature that reports a correlation between walking distance during the 6MWT and MSWS score of *r* = −0.81. Similarly, both ground truth and estimated speeds are significantly, and similarly, correlated with EDSS_SR_ score (estimated: *r* ≤ −0.72,*p* < 0.01, ground truth: *r* = −0.79,*p* < 0.01) as well. These results also agree well with existing literature that reports a correlation between walking distance during the 6MWT and EDSS score of *r* = −0.73 [5]. Lastly, there is a relationship between speed and if the subject reports falling in the last 6 months. Specifically, the comfortable walking speeds for subjects who have fallen in the last six months are 0.22 m/s slower than those who have not where models estimate differences between 0.19 and 0.25 m/s. The directionality of these results agree well with existing literature examining the relationship between walking speed and fall history [7]. These results clearly demonstrate that the proposed technique for estimating walking speed is able to preserve the relationship between the true speed and clinical assessments of MS symptom severity (EDSS_SR_ and MSWS) and fall history.

The primary limitation of this study is that results are based on data collected during treadmill walking. As a result, the performance may not generalize to ambulation in naturalistic environments. Future studies should explore changes in performance that result from employing the proposed technique for estimating walking speed during over-ground ambulation in subjects with and without MS-induced gait impairments. The study presented herein explores how models trained on subjects with unimpaired gait generalized to those with MS-induced gait impairments. A future direction could also focus on developing methodologies for the training of individualized models for each MS patient. This type of personalized approach, enabled by the BioStampRC technology, could improve the reported results further.

## Conclusion

In this study, we characterize the accuracy of walking speed estimations enabled by the combination of machine learning and the BioStampRC system, which includes a new skin-mounted, conformal wearable sensor, in subjects with and without MS-induced gait impairments. Within each group, we analyze the relationship between estimation accuracy and device location combinations. Finally, we assess the ability of the resulting walking speed estimations to maintain the clinical relevance of the ground-truth values. Specifically, we establish the accuracy of this technique on treadmill walking data sampled from subjects with normal gait patterns and find that accuracy improves with more distal device locations and the inclusion of additional devices, with the best model systematically overestimating speed by only 0.01 m/s, detecting changes in speed to within less than 1%, and achieving a root-mean-square-error of 0.12 m/s. We then assess the performance of models trained on normal subjects in a population of subjects with MS-induced gait impairments, and find that accuracy improves with proximal locations, and the inclusion of additional devices. The best performing model systematically overestimates speed by 0.01 m/s, quantifies changes in speed to within 1%, and achieves a root-mean-square-error of 0.14 m/s. We further demonstrate that the estimated walking speed maintains the clinical significance of the ground truth speed in this population, where the ground truth and estimated speeds during comfortable walking tests are highly correlated with impairment severity as quantified by EDSS_SR_ (*r* ≤ −0.72) and MSWS (*r* ≤ −0.84) scores. These results support the extension of accelerometer-based models for estimating walking speed developed on data from normal subjects to MS patients with gait impairments. Based on the results presented herein, and the wearable nature of the BioStampRC devices, this approach points toward the future ability to monitor parameters that characterize mobility impairment, such as gait speed, outside the clinic.

## Supporting information

S1 Appendix Validation of treadmill speed. (DOCX)

S1 File Features and truth speeds from healthy subjects. (ZIP)

S2 File Features and truth speeds from MS patients. (ZIP)

S3 File Estimated and truth speeds from healthy subjects and MS patients. (ZIP)
