# Supplementary material for: A machine learning approach for gait speed estimation using skin-mounted wearable sensors: From healthy controls to individuals with multiple sclerosis
Source: PLoS One. 2017 Jun 1;12(6):e0178366. doi: 10.1371/journal.pone.0178366 (PMC5453431; doi:10.1371/journal.pone.0178366)
Supplement: S1 Appendix — (DOCX) [file pone.0178366.s001.docx]

**S1 Appendix**

For treadmill activities, ground truth gait velocities were determined by each treadmill speed used. As a means to validate the efficacy of speed measurements reported by the treadmill, before starting the study, author NM performed each treadmill activity while simultaneously tracking the distance traveled by the treadmill belt using a perambulator. The measured distance was then used to calculate a true treadmill speed (measured distance / activity duration), which is shown, relative to the speed reported by the treadmill in Table 4. This test demonstrates that the treadmill used for Protocol A of this study accurately reports walking speed.

**S1 Table. Comparison of reported and measured treadmill speed.**

| **Treadmill Speed (m/s)** | **Measured Speed (m/s)** |
| --- | --- |
| 0.50 | 0.49 |
| 0.75 | 0.76 |
| 1.00 | 0.99 |
| 1.25 | 1.26 |
| 1.50 | 1.50 |
